# Reasons physicians do not recommend and patients refuse adjuvant chemotherapy for stage III colon cancer: a population based chart review

**DOI:** 10.1186/1756-0500-5-269

**Published:** 2012-06-07

**Authors:** Mohamed El Shayeb, Andrew Scarfe, Yutaka Yasui, Marcy Winget

**Affiliations:** 1 Department of Public Health Sciences, School of Public Health, University of Alberta, 3-300 Edmonton Clinic Health Academy, 11405-87th Avenue NW, Edmonton, AB, Canada, T6G 1 C9; 2 Department of Oncology, Faculty of Medicine & Dentistry, University of Alberta, CCI, 11560 University Avenue, Edmonton, AB, Canada, T6G 1Z2; 3 Cross Cancer Institute, Alberta Health Services, 11560 University Avenue, Edmonton, AB, Canada, T6G 1Z2; 4 Community Oncology, Cancer Care, Alberta Health Services, 1500- 10123 99 Street, Edmonton, AB, Canada, T5J 3 H1

## Abstract

**Background:**

Surgery followed by adjuvant chemotherapy has been the standard of care for the treatment of stage III colon cancer since the early 1990’s. Despite this, large proportions of patients do not receive adjuvant chemotherapy. We aimed to identify physicians’ and patients’ reasons for treatment decisions.

**Methods:**

A retrospective population based study was conducted that included all surgically treated stage III colon cancer patients diagnosed in Alberta between 2002 and 2005 who had an oncologist-consult to discuss post-surgical treatment options. Patient demographics and stage were obtained from the Alberta Cancer Registry. Chart reviews were conducted to extract treatment details, the oncologists’ reasons for not recommending chemotherapy, and patients’ reasons for refusing chemotherapy. The number and proportion of patients who were not recommended or refused chemotherapy were calculated.

**Results:**

A total of 613 patients had surgery followed by an oncologist-consult. Overall, 168 (27%) patients did not receive chemotherapy. It was not recommended for 111 (18%) patients; the most frequent reason was presence of one or more co-morbidities (34%) or combination of co-morbidity and age or frailty (22%). Fifty-eight (9%) patients declined chemotherapy, 22% of whom declined due to concerns about toxicity.

**Conclusion:**

Some co-morbidities are clinical indications for not receiving adjuvant chemotherapy, however, the high percentage of patients who were not recommended adjuvant chemotherapy due to co-morbidities according to clinical notes but who had a low Charlson co-morbidity score suggests variation in practice patterns of consulting oncologists. In addition, patients’ reasons for refusing treatment need to be systematically assessed to ensure patients’ preferences and treatment benefits are properly weighed when making treatment decisions.

## Background

Treatment guidelines for stage III colon cancer that include post-surgical (adjuvant) chemotherapy have existed for twenty years [1]. They are based on the results of clinical trials that compared adjuvant chemotherapy following surgery versus surgery alone [2-4]. Clinical trials demonstrated a relative risk reduction of 40% for recurrence and 33% for mortality [3]. Despite these evidence-based treatment guidelines, several studies conducted in the United States and Canada have shown that a large proportion of patients with stage III colon cancer do not receive adjuvant chemotherapy [5-11].

The healthcare system in the province of Alberta, Canada, is publicly funded and administered; standard treatments and corresponding visits to specialists are free. Treatment for cancer patients, other than surgery, is organized and coordinated provincially. In order to receive adjuvant chemotherapy, a cancer patient must first have a consultation with an oncologist to discuss treatment options. During the consultation visit, the oncologist may not recommend chemotherapy and/or the patient may refuse the recommended treatment. Reasons chemotherapy may not be initiated, therefore, are: 1) the patient was not referred to an oncologist; 2) the patient did not attend the consultation after being referred; 3) the oncologist did not recommend chemotherapy; or 4) the patient refused the recommended chemotherapy. There are six cancer facilities that offer oncologist-consults and about 25 oncologists conduct almost all the consultations for colorectal cancer patients.

We recently reported [11] that 20% of patients diagnosed with stage III colon cancer did not have a consultation with an oncologist to discuss adjuvant chemotherapy after surgical removal of their tumor. Thirty-seven percent of those who had a consultation did not receive adjuvant chemotherapy within 12 weeks of surgery, as recommended by treatment guidelines [12]. Older age, co-morbidities, region of residence, and median household income were significantly associated with not having a consultation, while older age and co-morbidities were associated with not receiving adjuvant chemotherapy [11]. Here we report a follow-up to that study, a population-based chart review on all patients who had a consultation with an oncologist, in order to: 1) determine the proportion of patients who did not receive chemotherapy due to the oncologist not recommending treatment and the proportion of patients who refused the treatment and 2) identify and describe the reasons the oncologists gave for not recommending adjuvant chemotherapy, rather than just associated factors, and the patients’ reasons for refusing it.

## Methods

### Study population

All residents of Alberta who were diagnosed with histologically confirmed stage III colon adenocarcinoma in years 2002 to 2005 were identified from the Alberta Cancer Registry. The Alberta Cancer Registry, initiated in 1942, is a member of the North American Association of Comprehensive Cancer Registries and has a proven record of high quality data [13]. Stage was based on the American Joint Committee on Cancer Tumor, Node, and Metastasis (AJCC TNM) staging [14]. Stage III colon cancer refers to cancer that has spread to the regional lymph nodes but not spread to distant organs. Patients were included if they had surgical removal of the tumor followed by a consultation with an oncologist to discuss post-surgery treatment options. Patients were excluded if they were treated outside of the province or had another cancer within 6 months of their colorectal cancer diagnosis.

Date of consultation with an oncologist to discuss post-surgical treatment options was obtained from electronic cancer medical records, which have been in place since 2002. Based on previous quality assurance activities, all consultations within six months of diagnosis were initially identified to be a potential consultation to discuss post-surgical treatment options [15]. Progress notes for all patients who did not have a consultation within 6 months of diagnosis were also reviewed to ensure that all patients with appropriate consultations were included. Although available electronically, progress notes are based on dictations which are not standardized.

The study was reviewed and approved by Alberta Cancer Board Research Ethics Board.

### Chart review

A chart review was conducted to extract information related to the discussion of adjuvant chemotherapy and the ultimate treatment decision. Specifically, the following information was abstracted: 1) whether the physician recommended chemotherapy; 2) the reason(s) the oncologist did not recommend chemotherapy; 3) whether the patient initiated chemotherapy; and 4) the reason(s) patients refused chemotherapy. The reasons for not recommending and for refusing chemotherapy were identified *a priori* and are listed in Tables 1 and 2. The definitions and rules used for selecting each reason are also listed. To ensure consistency in data extraction and interpretation, three reviewers (ME, AS, MW) independently abstracted a random sample of 50 charts. Answers were compared and discrepancies discussed and resolved. The reasons for not recommending or refusing chemotherapy and their corresponding definitions were modified for clarification, as needed.

**Table 1:** Definitions of oncologists’ reasons for not recommending chemotherapy

**Reasons oncologists did not recommend adjuvant chemotherapy**	**Definition**
Medical complications of surgery	Any medical incident during or just following surgery (e.g. myocardial infarction, stroke)
Surgical complications of surgery	Any surgery related complication (e.g. delayed wound healing, wound infection, abscess)
Co-morbidities	A medical condition or chronic disease preceding the onset of cancer and perceived by oncologist as a contraindication for chemotherapy (e.g. coronary heart disease, heart failure, kidney or liver failure)
Frailty/Performance status	Generalized weakness or poor physical performance status
Pharmacological concern	Potential drug-drug interaction between the adjuvant chemotherapeutic regimens and other drugs a patient is using for a chronic or incidental medical condition. Potential complication or adverse event of chemotherapy in a susceptible patient (e.g. vasospasm in a patient susceptible to coronary heart disease)
No social network or support	Lack of social, emotional, or physical support
Age	Patient age was the reason or one of the reasons for not recommending chemotherapy
Disease progression	Progression of the cancer to stage IV by the time the patient had a consultation with the oncologist and/or prior to receiving post-surgical treatment
Other	Reasons stated other than those listed above (e.g. non-conclusive staging at the time of consult)
Unclear	Reason(s) for not recommending chemotherapy are vague and not clearly stated

**Table 2:** Definitions of patients’ reasons for refusing adjuvant chemotherapy

**Reasons patients refused adjuvant chemotherapy**	**Definition**
Toxicity concerns	A perceived potential toxicity side effect (e.g. hair loss, excessive nausea and vomiting).
Co-morbidities	A medical condition or chronic disease preceding the onset of cancer and perceived by patient as a barrier to tolerating chemotherapy (e.g. coronary heart disease, heart failure, kidney or liver failure)
Transportation	Difficulty reaching the treatment facility due to either unavailability of means of transportation or cost of transportation
Age	Patient age was the reason or one of the reasons for refusing chemotherapy (e.g. patient felt they have lived long enough, perceived limited life expectancy)
No social network or support	Lack of social, emotional, or physical support
Other	Reasons stated other than those listed above (e.g. fear of injections)
Unclear	Reason(s) for refusing chemotherapy are vague and not clearly stated

### Demographic factors and co-morbidity scores

In addition to being the primary source for identifying the study population, the Alberta Cancer Registry was also the source for the following demographic data: gender; age; region of residence at diagnosis; and year of diagnosis. The patient’s postal code at diagnosis was used to calculate the driving distance to the nearest cancer center using the driving direction link from the internet Yellow Pages^©^ (http://www.yellowpages.ca), as previously described [11]. Neighborhood-level socioeconomic data from the 2001 Canadian census were also obtained to describe the patient cohort. Variables included median annual household income, percentage of people living alone, percentage not graduated from high school, and percentage employed.

Modified Charlson co-morbidity scores were calculated using an updated version of the *Deyo et al.*[16] method that was developed by *Quan et al.*[17] Co-morbidity scores were calculated using diagnosis codes obtained from two provincial hospital databases: the Ambulatory Care Classification System and the Discharge Abstracts Database. These databases contain dates of visits/admissions/discharge, diagnosis codes, and procedure codes for all hospital outpatient and inpatient visits, respectively, in the province. Modified Charlson co-morbidity scores were calculated using diagnosis codes that occurred within one year prior to each patient’s colon cancer diagnosis; diagnosis coding systems used were the International Classification of Diseases version 9- Clinical Modification (ICD-9-CM) and the International Classification of Diseases version 10-Canada (ICDM-10-CA).

### Statistical analysis

The number and proportion of patients who refused, were not recommended or received adjuvant chemotherapy were calculated by the demographic characteristics included in the study. Chi-square tests were calculated to determine statistical differences in patient distributions across the three chemotherapy receipt/non-receipt categories. The number and proportion of patients who were not recommended chemotherapy by their oncologist or who refused chemotherapy were calculated by reason for not recommending or refusing adjuvant chemotherapy, respectively. Some reasons were combined due to small numbers.

The frequencies of the main reasons recorded in progress notes for not recommending chemotherapy by oncologists were cross-tabulated with patient age at diagnosis and co-morbidity scores. This allows comparison between the major stated reasons oncologists did not recommend adjuvant chemotherapy and the major factors found to be associated with not receiving adjuvant chemotherapy in our previous study [11] which included the same patient population. Analyses were conducted using Stata SE version 10, StataCorp., Texas, USA.

## Results

There were 722 residents of Alberta who were diagnosed with stage III colon adenocarcinoma in the years 2002 to 2005 of which 618 patients had a consultation with an oncologist to discuss post surgical adjuvant treatment. Following the chart review, five patients were excluded for the following reasons: two patients were treated outside of Alberta, two patients had rectal cancer, and one patient’s consultation was not related to post-surgical treatment. The analysis included the remaining 613 patients.

**Figure 1:**
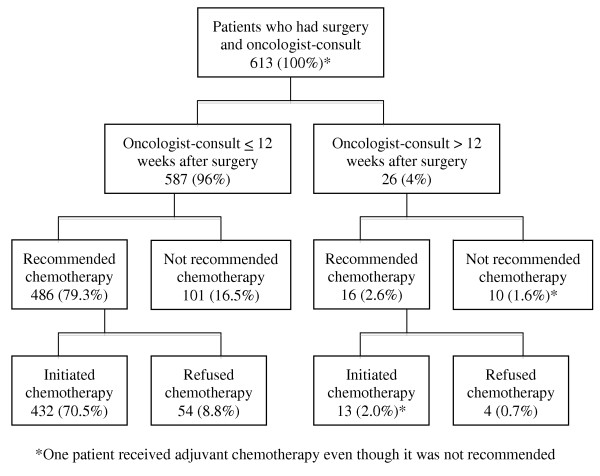
Proportion of patients with stage III colon cancer in Alberta who were not recommended and/or refused adjuvant chemotherapy.

Figure 1 shows the proportion of patients for whom chemotherapy was not recommended by their oncologist and the proportion of patients that refused adjuvant chemotherapy in relation to the timing of their consultation with respect to their surgery. Overall 27% (168) of the patients did not commence adjuvant chemotherapy. It was not recommended for 111 (18%) patients and 58 (9%) patients refused adjuvant chemotherapy. Only one patient decided to pursue adjuvant chemotherapy despite not being recommended by the oncologist. Of interest is that 26 patients had a consultation more than 12 weeks after their surgery, the maximum time that should elapse before initiating adjuvant chemotherapy as per treatment guidelines [12]; 16 of these patients were recommended chemotherapy and 12 of them initiated it. Delayed or late consult was not the reason for not recommending adjuvant chemotherapy to any of patients who had a consultation more than 12 weeks post-surgery.

Table 3 presents the distribution of patient characteristics by refusal, recommendation and receipt of chemotherapy. Older patients were less likely to be recommended chemotherapy and were more likely to refuse it if recommended (p < 0.0001). Patients in the northern regions of the province were more likely to refuse chemotherapy whereas those in the south were less likely to have it recommended (9 = 0.03). Chemotherapy recommendation and/or patient refusal were related to the neighborhood income (p = 0.01), percent living alone (p < 0.0001), and percent employed measures (p = 0.04). Patient gender, year of diagnosis, distance from a cancer center, and neighborhood education level were not associated with receipt/refusal/recommendation of chemotherapy.

**Table 3:** Relationship between refusal, recommendation, and receipt of adjuvant chemotherapy and characteristics of patients diagnosed with stage III colon cancer in Alberta between 2002 and 2005 who had an oncologist-consult

	**Refused Chemotherapy**		**Chemotherapy not Recommended**		**Received Chemotherapy**		**Total**	
**Patient Characteristics**	**n**	**(%)**^**1**^	**n**	**(%)**^**1**^	**n**	**(%)**^**1**^	**n**	**(%)**^**2**^
**Total**	**58**	**(9)**	**110**	**(18)**	**445**	**(73)**	**613**	**(100)**
**Gender:**	**P = 0.84**							
Female	27	(9)	48	(17)	208	(73)	283	(46)
Male	31	(9)	62	(19)	237	(72)	330	(54)
**Age at diagnosis:**	**P < 0.0001**							
<65	10	(4)	11	(4)	232	(92)	253	(41)
65-69	5	(7)	6	(8)	60	(85)	71	(12)
70-74	11	(11)	13	(13)	76	(76)	100	(16)
≥ 75	32	(17)	80	(42)	77	(41)	189	(31)
**Geographical regions:**	**P = 0.03**							
South	4	(9)	10	(22)	31	(69)	45	(7)
Calgary	8	(4)	40	(21)	143	(75)	191	(31)
Central	7	(7)	18	(19)	72	(74)	97	(16)
Edmonton	29	(13)	35	(16)	157	(71)	221	(36)
North	10	(17)	7	(12)	42	(71)	59	(10)
**Distance from cancer facility**	**P = 0.97**							
< 90 km	49	(10)	94	(18)	369	(72)	512	(84)
90-180 km	5	(8)	9	(15)	47	(77)	61	(10)
> 180 km	4	(10)	7	(18)	29	(73)	40	(7)
**Year of diagnosis:**	**P = 0.10**							
2002	8	(6)	26	(18)	109	(76)	143	(23)
2003	22	(13)	23	(14)	124	(73)	169	(28)
2004	17	(12)	26	(18)	100	(70)	143	(23)
2005	11	(7)	35	(22)	112	(71)	158	(26)
Median annual household income^*^	**P = 0.01**							
< $37881	14	(12)	29	(25)	75	(64)	118	(20)
$37881 – $51057	15	(10)	29	(19)	110	(71)	154	(26)
$51058 - $67124	19	(12)	29	(19)	107	(69)	155	(26)
> $67124	9	(5)	20	(12)	143	(83)	172	(28)
% Living alone^*^	**P < 0.0001**							
< 7%	20	(7)	36	(13)	218	(80)	274	(46)
7 – 24%	29	(19)	53	(19)	196	(71)	278	(46)
> 24%	8	(17)	18	(38)	21	(45)	47	(8)
**Patient Characteristics**	**n**	**(%)**^**1**^	**n**	**(%)**^**1**^	**n**	**(%)**^**1**^	**n**	**(%)**^**2**^
% Not graduated from high school^*^	**P = 0.50**							
< 27%	29	(9)	51	(16)	234	(75)	314	(52)
> 27%	28	(10)	56	(20)	201	(71)	285	(48)
% Employed^*^	**P = 0.04**							
< 60%	16	(10)	40	(25)	101	(64)	160	(27)
60 – 71%	18	(8)	36	(17)	160	(75)	211	(35)
> 71%	23	(10)	31	(14)	174	(76)	228	(38)

1 – Row percentage.

2 – Column percentage.

*14 patients had missing data for neighborhood-level variables based on 2001 Canadian census.

The recorded reasons in progress notes that oncologists did not recommend adjuvant chemotherapy to the 111 patients are presented in Table 4. The most common reason was the presence of co-morbidities (62 patients). Presence of co-morbidities was the only reason for not recommending chemotherapy to 38 (34%) patients and was combined with age, frailty, or another reason for 24 (22%) patients. Frailty and age, alone or combined, were reasons for not recommending adjuvant chemotherapy to 22 (19%) patients. Medical or surgical complications of surgery were the reason oncologists did not recommend adjuvant chemotherapy to 12 (11%) patients. Seven (6%) patients were not recommended chemotherapy due to disease progression.

**Table 4:** Physicians’ reasons for not recommending adjuvant chemotherapy to patients diagnosed with stage III colon cancer in Alberta between 2002 and 2005

**Single Reason**	**n**	**(%)**	**Multiple Reasons**	**n**	**(%)**
Co-morbidity only	38	(34)	Co-morbidity and age	11	(10)
Frailty only	8	(7)	Co-morbidity and frailty	7	(6)
Age only	6	(5)	Co-morbidity and other reason	6	(6)
Complication of surgery*	12	(11)	Age and frailty	8	(7)
Disease progression	7	(6)	Other - multiple reasons	3	(3)
Other - single reason	5	(5)			
Total	76	(68)	Total	35	(32)

*Either surgical or medical complication of surgery described in Table 1.

Fifty-eight (9%) patients refused adjuvant chemotherapy even though it was recommended; Table 5 presents the reasons for refusal. The most common recorded reason for refusing adjuvant chemotherapy by patients was toxicity concerns (13 patients), however, reason for refusal was unclear for over half the patients that refused (34 patients).

**Table 5:** Stage III colon cancer patients’ reasons for refusing adjuvant chemotherapy

**Reason for refusing chemotherapy**	**n** 58	**(%)** (100)
Unclear	34	(59)
Toxicity concerns	13	(22)
Age	4	(7)
Co morbidities	3	(5)
Other	4	(7)

Table 6 shows the results of the cross-tabulation between the frequency of the three main recorded reasons for not recommending adjuvant chemotherapy that were extracted from the patient charts (co-morbidity, age, and frailty) with the actual patient age at diagnosis and co-morbidity score calculated from administrative data; this comparison was done to see how well the factors of actual age and co-morbidity score corresponded to those factors as reasons for not recommending adjuvant chemotherapy. Of those patients for whom adjuvant chemotherapy was not recommended due to presence of co-morbidity according to patient charts, 22 of 62 patients (35%) had a co-morbidity score of 0 calculated from the administrative data. Conversely, 94 (19%) of the 502 patients for whom chemotherapy was recommended had a co-morbidity score of 2 or more. 44 (70%) of the patients who were not recommended adjuvant chemotherapy due to co-morbidities were 75 years or older at the time of their diagnosis. All of the patients who were not recommended adjuvant chemotherapy due to age were 75 years or older. Similarly, 21 of the 24 “frail” patients were 75 years or older.

**Table 6:** Agreement between primary reasons for not recommending chemotherapy extracted from patient charts vs. patient age and Charlson co-morbidity index

**Oncologists’ reasons for not recommending adjuvant chemotherapy**							
	Co-morbidities N = 62 (100%)		Age N = 25 (100%)		Frailty N = 24 (100%)		Total
**Charlson comorbidity index**							
0	22	(35)	15	(60)	11	(46)	48
1	14	(23)	5	(20)	6	(25)	25
2 or more	26	(42)	5	(20)	7	(29)	38
**Age**							
<65	6	(10)	0		0		6
65-69	3	(5)	0		0		3
70-74	9	(15)	0		3	(13)	12
≥ 75	44	(70)	25	(100)	21	(87)	90

## Discussion

Previous studies that have evaluated adherence to treatment guidelines for stage III colon cancer and barriers to receiving them have used administrative data or surveys [5,7-11,18-21]. These study designs identify associations but not causality. We conducted a chart review to identify the documented reasons or causes for non-receipt of adjuvant chemotherapy rather than associated independent variables or factors.

Our results show that two thirds of the patients who did not initiate chemotherapy did not because their oncologist did not recommend it to them; the primary reasons recorded in patient charts were co-morbidity followed by age and frailty, respectively. The remaining third refused the treatment despite being recommended. Other studies have found that the oncologist’s recommendation is the main factor determining whether or not a patient receives chemotherapy and that patients tend to follow their oncologist’s recommendation regarding adjuvant chemotherapy [22,23]. Patient preferences and their perception towards their outcomes and survival probabilities also have a large impact on decision-making [24].

22% of patients refused chemotherapy due to toxicity concerns. More importantly, there was not a clear reason for refusal in 59% of the patients who declined chemotherapy. These patients tended to leave the consultation visit undecided and ask their oncologist for time to consider their options. Ultimately, they either never replied (even when phone messages were left by nursing staff) or called to decline without giving specific reasons. We found that a chart review, therefore, was not an effective way of identifying reasons for patient refusal of treatment. Patient surveys or interviews are likely more efficient, however, they are challenging, as cancer patients are often asked to complete surveys for various purposes (e.g., patient satisfaction, research studies); it is important not to overly burden these ill patients. Another challenge with patient surveys is the selection bias based on the degree of illness in the responders so the results may not be generalizable.

Co-morbidity alone or combined with other reasons was the most frequently documented reason patients were not recommended adjuvant treatment in our study, however, presence of co-morbidities, such as heart disease, did not universally preclude patients from being recommended to receive adjuvant chemotherapy. Two recent large, geographically diverse, surveys conducted among surgical and medical oncologists examined how different hypothetical clinical scenarios, varying patient age and co-morbidities, influence the recommendation of adjuvant chemotherapy for patients with stage III colon cancer [18,19]. The surveys found that physicians agree on recommending chemotherapy for healthy young individuals, but differ widely for older patients with or without co-morbidities; the recommendations differed also by the severity of the co-morbidity (mild, moderate, or severe) regardless of patient age. Consistent with these surveys, our results showed inconsistency in recommendations based on patient age and presence of co-morbidities. Adjuvant chemotherapy, however, provides a significant survival benefit for colon cancer patients with chronic diseases including heart failure, chronic obstructive pulmonary diseases, and diabetes [25]. Efforts are needed to improve consensus amongst oncologists on the value of adjuvant chemotherapy in currently under-treated patient groups, particularly those with co-morbidities.

To further investigate the way physicians integrate the presence of co-morbidities and its interaction with age in making a treatment recommendation, we cross-tabulated the frequency of these reasons extracted from our chart review to assess the degree to which actual age and co-morbidity scores corresponded to them. The co-morbidity index (a tool that has been validated as a reliable index in predicting one-year survival) [16,17] was found to be 0 in 22 patients for whom co-morbidity was the reason for not being recommended adjuvant chemotherapy. On the flip side, 94 patients for whom chemotherapy was recommended had a co-morbidity index of 2 or more, indicating the significant variation amongst oncologists in Alberta in their perception of the importance of co-morbidities in recommending adjuvant chemotherapy. A limitation to this comparison is that we did not extract details on co-morbidities that patients had regardless of whether they were recommended adjuvant chemotherapy. This would have allowed for a more direct comparison to the co-morbidity scores as well as better assess variation in practice based on specific co-morbidities and their severity.

The discrepancy between chart review data and administrative data can also be attributed to the way in which the co-morbidity score is calculated. Diagnoses for 17 conditions that are known to increase the risk of death in the year following hospitalization are included in the calculation of the co-morbidity score. If a patient was not seen in a hospital as an inpatient or an outpatient in the year prior to their diagnosis, no co-morbidities would be identified; similarly, if a patient was seen in a hospital for reasons unrelated to the co-morbidities used to calculate the co-morbidity score, for instance, a broken leg, it is possible that all existing co-morbidities would not be coded in the visit.

Two population-based studies have shown a survival advantage in standard clinical practice in patients with stage III colon cancer treated with chemotherapy within 12 weeks of surgery; co-morbidity was not significantly associated with survival in one of them [26], however, it was associated with survival in the other one [27].

## Conclusion

Despite the fact that most studies that have evaluated adherence to treatment guidelines for patients with stage III colon cancer have found that patient age is the factor most strongly associated with not receiving adjuvant chemotherapy, presence of one or more co-morbidities is the primary documented reason patients with stage III colon cancer are not recommended to receive adjuvant chemotherapy in Alberta, Canada. Given the large percentage of patients who did not receive adjuvant treatment, the inconsistency of whether chemotherapy was recommended to patients with co-morbidities, and the poor correlation between the co-morbidity score and co-morbidity as the reason adjuvant chemotherapy was not recommended, further studies and discussion to better define patients who are clinically appropriate to receive adjuvant chemotherapy are needed. Specifically, studies that evaluate the relationship between survival, adjuvant chemotherapy and co-morbidities in patients with stage III colon cancer are needed to inform development of clearer evidence-based treatment guidelines and/or improve consensus amongst oncologists.

## Authors’ contributions

MW and ME conceptualized the study idea and design, validated the chart review and edited the manuscript. MW was responsible for overseeing the study and reviewed the manuscript. YY conceptualized the statistical analysis plan and shared in editing and reviewing the manuscript. AS conceptualized the reasons for not recommending and declining the treatment, functioned as primary consultant for clinical data issues during data extraction process, shared in the validation process of chart review, editing and reviewing the manuscript. ME carried out the data extraction and data entry process, implemented the statistical analysis plan, prepared the draft manuscript, integrated other authors’ reviews, and prepared the final manuscript. All authors read and approved the final manuscript.

## Competing interests

All authors declare that they have no financial or non-financial competing interest.
